# Human Brain Organoid: A Versatile Tool for Modeling Neurodegeneration Diseases and for Drug Screening

**DOI:** 10.1155/2022/2150680

**Published:** 2022-08-25

**Authors:** Cuili Ma, Hwanwook Seong, Xiaowei Li, Xiao Yu, Shunliang Xu, Yujing Li

**Affiliations:** ^1^Department of Internal Medicine, Qingdao Agricultural University Associated Hospital, Qingdao 266109, Shandong Province, China; ^2^Department of Human Genetics, Emory University School of Medicine, 615 Michael St, Atlanta, GA30322, USA; ^3^School of Life Sciences, Nanjing University, 163 Xianlindadao Rd, Nanjing 210023, China; ^4^Department of Neurology and Department of Nutrition, The Second Hospital Cheeloo College of Medicine, Shandong University, 247 Beiyuan Road, Jinan 250033, Shandong Province, China; ^5^Department of Neurology, The Second Hospital Cheeloo College of Medicine, Shandong University, 247 Beiyuan Road, Jinan 250033, Shandong Province, China

## Abstract

Clinical trials serve as the fundamental prerequisite for clinical therapy of human disease, which is primarily based on biomedical studies in animal models. Undoubtedly, animal models have made a significant contribution to gaining insight into the developmental and pathophysiological understanding of human diseases. However, none of the existing animal models could efficiently simulate the development of human organs and systems due to a lack of spatial information; the discrepancy in genetic, anatomic, and physiological basis between animals and humans limits detailed investigation. Therefore, the translational efficiency of the research outcomes in clinical applications was significantly weakened, especially for some complex, chronic, and intractable diseases. For example, the clinical trials for human fragile X syndrome (FXS) solely based on animal models have failed such as mGluR5 antagonists. To mimic the development of human organs more faithfully and efficiently translate in vitro biomedical studies to clinical trials, extensive attention to organoids derived from stem cells contributes to a deeper understanding of this research. The organoids are a miniaturized version of an organ generated in vitro, partially recapitulating key features of human organ development. As such, the organoids open a novel avenue for in vitro models of human disease, advantageous over the existing animal models. The invention of organoids has brought an innovative breakthrough in regeneration medicine. The organoid-derived human tissues or organs could potentially function as invaluable platforms for biomedical studies, pathological investigation of human diseases, and drug screening. Importantly, the study of regeneration medicine and the development of therapeutic strategies for human diseases could be conducted in a dish, facilitating in vitro analysis and experimentation. Thus far, the pilot breakthrough has been made in the generation of numerous types of organoids representing different human organs. Most of these human organoids have been employed for in vitro biomedical study and drug screening. However, the efficiency and quality of the organoids in recapitulating the development of human organs have been hindered by engineering and conceptual challenges. The efficiency and quality of the organoids are essential for downstream applications. In this article, we highlight the application in the modeling of human neurodegenerative diseases (NDDs) such as FXS, Alzheimer's disease (AD), Parkinson's disease (PD), and autistic spectrum disorders (ASD), and organoid-based drug screening. Additionally, challenges and weaknesses especially for limits of the brain organoid models in modeling late onset NDDs such as AD and PD., and future perspectives regarding human brain organoids are addressed.

## 1. Introduction

Due to the inaccessibility of human organs/tissues/systems, biomedical studies on human development and disease pathophysiology are conducted only in animal models or cell-based in vitro assays. Animal models, particularly mouse models, have contributed significantly to gaining insight into the developmental and pathophysiological understanding of human diseases. However, the large evolutionary distance between mice and humans led to a discrepancy in genetic, anatomic, and physiological aspects between animal models and humans. This makes the existing animal models unable to efficiently simulate the development of human organs and systems at the anatomical and pathophysiological levels. Given that clinical trials serve as the fundamental prerequisite for human disease therapy, all the clinical trials designed and conducted on animal models significantly weaken the translational efficiency from the basic biomedical research outcomes to clinical therapy. To mimic the development of human organs more faithfully and facilitate translational efficiency from benchwork to clinical trials, in vitro organoid models derived from the stem cells have been established as a more effective and reliable platform relative to animal models. Organoids are characterized as miniaturized and simplified versions of an organ generated in vitro but could recapitulate key features of human organ development. As such, organoids pave a novel avenue for in vitro human disease models, an invaluable platform for biomedical studies and pathological investigation of human diseases. Meanwhile, human organoids have been applied for drug screening and successful achievements have been made. Drug development has been acknowledged as the key issue for advancing clinical therapy, particularly for diseases such as cancer, heart diseases, and neurological disorders. Therefore, a rapid and reliable drug screening strategy is essential to identify “hit” compounds prior to assessing the metabolic and toxicologic mechanisms both in vitro and in vivo. To develop an efficient operating system for primary screening, the cell-based efficacy and toxicity assay has been extensively used. However, the cell-based drug assays fail to recapitulate the response of human organs or systems to the compound agents, leading to a large-scale loss of resources in drug discovery and a low rate of successful cases.

As the headquarter of the human body, the brain, structurally composed of a complex architecture, performs cognitive functions to orchestrate the normal functions of all the systems and organs via extraordinarily intricate networks [1–3]. Developmental abnormality in the structure of the brain could lead to severe neurological or psychiatric disorders. While the limited access to primary patient brain tissues provides the main source of gaining insight into disease pathology, the information it represents contributes to an understanding of consequential mechanisms [4]. Vertebrate animal models such as mice provide important resources for the dissection of the developmental mechanisms and pathogenesis of disorders [5]. However, when we evaluate the significance of the discovery made in the mouse for translation to clinical application, it is important to remember the dramatic evolutionary distance between levels of mammals and humans as well as the subtle differences of the nervous system in both morphology and complexity [6]. It is plausible to say that the animal models fail to recapitulate the numerous key features unique to the development of the human nervous system and neurological disorders.

Central nervous system (CNS) diseases are much more complicated relative to other organ disorders, leading to lower efficiency for modeling CNS diseases via animal models. Faithfully mimicking the human brain's development, function, and susceptibility to disease by using organoids could significantly enhance translation efficiency from biomedical studies to clinical trials [7, 8]. Architecturally, the 3-D brain organoid consists of a majority of all known human brain cell types such as progenitor, neuronal and glial cells [9–14].

Thus far, the pilot breakthrough has been made in the generation of numerous types of organoids representing different human organs as reviewed recently (Table 1) [15–20]. These brain organoids could partially recapitulate some aspects of human brain genesis and development, potentially modeling developmental neurological disorders, such as FXS, Alzheimer's disease (AD), Parkinson's disease (PD), and autistic spectrum disorders (ASD) (Table 2). In addition, brain organoids were also employed to model Schizophrenia [21, 22], Down syndrome [23], Lissencephaly [24–27], Rett syndrome [28], and Timothy syndrome [29]. Brain organoids have also been utilized for modeling parental alcohol and drug abuse [30, 31]. The organoids generated from the patient with Niemann-Pick disease type C (NPC), a neurodegenerative lysosomal storage disorder caused by genetic mutations could mimic the phenotype of the NPC patients [30]. Some cortical organoids were used for modeling the impairment of molecular subtype specifications caused by ectopically activating cellular stress pathways under the conditions of cell stress [32]. Some of the brain organoids were used for modeling the key features of ionizing radiation-induced DNA damage in human neurons to understand the repair mechanisms [33]. Some patient-derived glioma cerebral organoids have been developed for disease modeling to understand glioma biology and predict responses to chemotherapy drugs [34–37]. In this paper, we focus on the recent advances in human brain organoid models for neurological disorders including AD, PD, ASD, FXS as well as organoid-based drug screening. Additionally, we also discuss the challenges, weaknesses, and future perspectives of organoid research.

## 2. Brain Organoid-Based Modeling of Neurological Disorders

### 2.1. Modelling of AD Using Brain Organoids Derived from AD Patients

AD is a late-onset (at age over 65) disorder that is not caused by natural aging. It accounts for 60-80% of dementia cases associated with progressive memory loss and other cognitive abilities. Pathologically, AD is characterized by the accumulation of protein aggregates, tau plaques, and synaptic dysfunctions. Relative to cell and animal models, human cerebral organoids can efficiently mimic the key features of the human brain. Therefore, many *in vitro* organoids models have been developed for AD modeling ([38–40] [37, 41–45].

**Figure 1:**
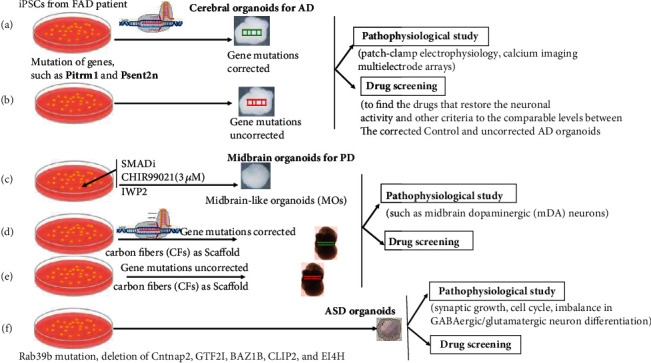
The AD, PD, and ASD isogenic organoids derived from the patient iPSCs where their mutated genes were corrected via CRISPR-CAS9 based genome editing as well as from the iPSCs where their mutated genes were uncorrected. These isogenic organoids could recapitulate the key pathophysiological features. A-B) iPSCs and the organoids from FAD patients. a) Gene mutation was corrected via CRISPR-CAS9 based genome editing in the iPSCs derived from FAD patients and the organoids from the FAD iPSCs; b) Both iPSCs and the organoids are identical except for the uncorrected gene mutation. C-E) iPSCs and organoids from PD patients. c) mid-brain-like organoids (MOs) generated with the addition of SMADi, CHIR99021 at 3uM, and IWP2 in the culture medium; d) Gene mutation was corrected via CRISPR-CAS9 based genome editing in the iPSCs derived from PD patient and the organoids from the PD iPSCs; e) Both iPSCs and the organoids are identical except for the uncorrected gene mutation. The organoids were generated using the carbon fibers (CFs) as scaffolds in both D) and E). f) iPSCs and the organoids from ASD patients with Rab39b mutation, deletion of Cntnap2, GTF2I, BAZ1B, CLIP2, and EI4H, but no mutation corrected organoids were available currently. The panels described the key features of the organoids, and the panels showed the potential pathophysiological study and drug screenings.

Most brain organoids for AD modeling harbor familial patient-specific genomic backgrounds including mutations, deletions, and insertions. Importantly, most of these AD patient-derived brain organoids are familial isogenic lines and are matched with normal brain organoids to serve as controls (Figures 1(a) and 1(b)). These organoids could partially recapitulate the key pathological features of the AD patient's brain on a molecular, cellular, and network-connectivity basis. Therefore, these organoids function as pilots for understanding the pathophysiological mechanisms on a patient-specific basis. Furthermore, these organoids could be employed as a platform for drug screening. It is highly expected that the drugs identified and validated to increase neuronal activity could contribute to therapy based on the patient-specific personalized medicine. The advances in AD organoid models within the recent two years are highlighted below.

#### 2.1.1. *PSEN2N* Mutation

The self-organizing cerebral organoids were generated from a familial AD patient with *PSEN2N* mutation and control organoids from an identical genetic background without *PSEN2N* mutation by genome editing. Treatment of these organoids with drugs that increase neuronal activity could facilitate the synchronization of high-frequency networks bursting at a comparable level in both control and AD organoids. Thus, these organoids can potentially become promising tools for AD pathological studies and a platform for drug screening [46].

#### 2.1.2. *BIN1* Gene Mutation

The *BIN1* KO organoids displayed the phenotype of early endosome narrowing, which could be rescued by the expression of BIN1iso1 but not BIN1iso9. Given that BIN1iso1 overexpression could enlarge the early endosomes and lead to neurodegeneration in human induced neurons (hiNs), it is plausible to suggest that the AD susceptibility gene *BIN1* could become an early biomarker for AD pathology [47].

#### 2.1.3. Mitochondrial Protease PITRM1-KO

Cerebral organoids derived from Pitrilysin metallopeptidase 1 (PITRM1)-KO iPSCs could recapitulate the pathological features of AD, such as the accumulation of protein aggregates, tau plaques, and synaptic dysfunctions. PITRM1 is a mitochondrial protease, and its deficiency causes a slow-progressing neurological disorder with a similar syndrome to AD, linking the mitochondrial function to the pathogenesis of common neurodegeneration [48].

#### 2.1.4. Mouse IFITM3-KO

Inflammatory cytokines induce the expression of IFITM3, a *γ*-secretase in neurons and astrocytes, which bind to *γ*-secretase and upregulate its activity, thereby increasing the production of amyloid-*β*. The expression of IFITM3 is increased with aging and in mouse models that express familial AD genes. Furthermore, knockout of IFITM3 reduces *γ*-secretase activity and the subsequent formation of amyloid plaques in a transgenic mouse model (5xFAD). The IFITM3 protein is upregulated in tissue samples from a subset of late-onset AD patients who exhibit higher *γ*-secretase activity. The quantity of IFITM3 in the *γ*-secretase complex possesses a strong positive correlation with *γ*-secretase activity in the late-onset AD patient samples. This discovery suggests that *γ*-secretase is modulated by neuroinflammation via IFITM3, thereby increasing the risk for AD pathogenesis [49].

#### 2.1.5. Mimicking Blood-Brain Barrier (BBB) Breakdown

To simulate the serum exposure consequence of BBB breakdown in AD patients, brain organoids from sporadic AD patients were exposed to human serum. AD-like pathologies were observed, such as magnified A*β* aggregates and elevated phosphorylated p-Tau levels, synaptic loss, and neural network damage [50].

#### 2.1.6. Spatiotemporal Expression of Tau

Given the significant contribution of tau to the pathogenesis of AD, the spatiotemporal expression of tau has been mapped during brain development using iPSC-derived cortical organoids. While tau expression was detected in radial glia, neuronal maturation led to the dramatic elevation of tau mRNAs by using single-cell RNA sequencing, RNA in situ hybridization, and IHC. Spatially, low expression levels were observed in SVZ radial glia and deep white matter intermediate progenitors. This discovery could pave the way for further studies on the pathophysiological mechanisms of triggering and enhancing tau expression, simplifying the identification of therapeutic targets for tauopathy-based neurodevelopmental disorders [51].

#### 2.1.7. BACE2 Mutation

Control or the BACE2 loss of function mutation (BACE2G446R) human brain organoids were used to investigate the contribution of BACE2 to AD pathogenesis. BACE2 was predominantly expressed in the ventricular zone and cortical plate of the organoids, and the expression levels were gradually elevated during the maturation of organoids. Furthermore, compared to control organoids, the mutant organoids displayed significantly enhanced apoptosis and elevated levels of A*β* oligomers, representing the AD-associated phenotypes [52].

#### 2.1.8. *ε*4/*ε*4 Genotype

The cerebral organoids generated from iPSCs derived from APOE *ε*3/*ε*3 or *ε*4/*ε*4 genotypes could recapitulate the APOE4-related phenotypes. To be specific, significant apoptosis and detrimental synaptic dysfunction were detected in the AD patient organoids. Furthermore, elevated A*β* and phosphorylated tau levels relative to the healthy subject-derived cerebral organoids were detected. Accordingly, conversion of APOE4 to APOE3 partially reversed the APOE4-associated phenotypes in cerebral organoids from AD patients. Molecularly, enhanced stress granules and irregular genes were linked to AD phenotypes. Thus, it could be inferred that the APOE4 may contribute to late-onset AD pathogenesis [53].

#### 2.1.9. Tau P301S Mutation

A new method was established recently for the generation of isogenic cerebral organoids for modeling AD with controlled genetic variables and mutation(s) in a specific gene by using an episomal plasmid vector derived from the Epstein-Barr virus (EBV). It turns out that this vector-based method could facilitate the easy and powerful generation of the isogenic cerebral organoids by avoiding the complexity and incompatibility offered by conventional genetic engineering and the CRISPR-Cas9 technology. More importantly, the isogenic cell lines generated from wild-type tau versus its mutant harboring the genetic form P301S were stable for more than 30 passages in terms of genetic and pathophysiological features. Thus, this strategy could make the generation of isogenic organoids easy and robust, facilitating the study of disease pathology, personalized medicine, and drug screening for clinical therapy [54].

#### 2.1.10. Mutations of PSEN1 M146V, APP^swe^, and PSEN1 *Δ*E9

In a separate study, organoids harboring familial AD mutations against their wild-type (WT) isogenic controls were employed as a platform for drug screening to identify drugs functional for therapy of hyperexcitability, subsequent extensive synapse loss, and cognitive dysfunction. The physiological assays based on this platform led to the identification of NitroSynapsin, a dual-allosteric NMDAR antagonist, that could eradicate the hyperactivity and rebalance the aberrant neural networks. Thus, this platform could be promising for large-scale screening to identify drugs for therapy of hyperexcitability and synaptic damage in AD patients [55].

#### 2.1.11. Histone Deacetylase-6 Inhibitor Partially Reverses the Phenotype of AD Organoids

Treatment of AD animal model (ADLP^APT^) brains and AD patient-derived brain organoids with CKD-504, a histone deacetylase-6 (HDAC6) inhibitor, could significantly degrade pathological tau plaques. Mechanistically, the inhibitor CKD-504 leads to the enhanced acetylation of tau, thereby recruiting chaperone proteins such as Hsp40, Hsp70, and Hsp110 to form complexes. The acetylated complexes with HSPs could bind to UBE2O and RNF14, the novel tau E3 ligases, degrading pathological tau via proteasomal pathways. This discovery could be translated into a clinical therapy for AD [56].

### 2.2. Modeling of PD Using Midbrain Organoids Derived from PD Patients

PD is another complicated progressive nervous system disorder. Almost all the PD modeling information originated from animal models before human brain organoids were available. Recently, midbrain organoids have been generated by improving the conventional strategy [57–61]. These midbrain organoids are of significant interest for modeling PD as they generate dopaminergic neurons expressing markers of Substantia Nigra identity, the most vulnerable to degeneration [62]. Studies showed that PD organoids could catch the key pathophysiological features of PD, suggesting their potential for pathological study and drug screening to identify the compounds for clinical therapy (Figures 1(c)–1(e)).

#### 2.2.1. Organoids from the Idiopathic Form of PD Patients

The first midbrain organoids were generated from iPSCs of the idiopathic form of PD patients, reprogrammed with the aid of non-integrating Sendai viral vectors. The mature organoids could simulate the expression of early and late neuronal markers as well as the statistical differences in the expression levels of these markers between the organoids from PD patients and healthy people. Therefore, it is highly expected that these organoids could be promising for modeling the idiopathic form of PD and in vitro pathological studies [57].

#### 2.2.2. The Isogenic Organoids Derived from Familial PD Patients

The isogenic midbrain organoids were derived from PD patients harboring a genomic mutation in LRRK2 G2019S and were employed for the pathogenic study. The key pathological features observed in the LRRK2-associated sporadic PD patient brains were also detected in the isogenic midbrain organoids. Molecularly, protein-protein interaction network assays have enabled the identification of TXNIP, a thiol-oxidoreductase, as a key contributor to the development of LRRK2-associated PD in the LRRK2 mutant organoids. Thus, these isogenic PD organoids provide a platform for pathological study as well as drug screening for clinical therapy of the LRRK2-associated sporadic PD [63]. More recently, human midbrain organoids derived from healthy individuals against their isogenic LRRK2-p.Gly2019Ser-mutant counterparts were compared to determine if the in vitro model simulates the in vivo equivalents from the aspects of developmental pathways and cellular events. It turns out that the midbrain organoids could model the early developmental stage of PD [64]. Midbrain organoids carrying the biallelic mutations of the *PINK1* gene from the patients and from the corrected cell lines by genome editing were employed for modeling PD. Compared to the corrected organoids, the patient organoids recapitulate the key PD features, consistent with the fact that mutation of the *PINK1* gene alone is sufficient to cause PD. Using both types of midbrain organoids as a platform, the selected compounds from the mouse model were tested [65]. In a separate study, human mid-brain-like organoids (hMLOs) harboring control or mutant DNAJC6 were generated to model the early-onset PD caused by a *DNAJC6* mutation. It turned out that the mutant hMLOs could recapitulate the key pathogenic features, thereby serving as a tool to investigate the pathology [66]. To decipher the role of the Bridging Integrator 1 (*BIN1*) gene in AD pathogenesis, the induced human cerebral organoids and neurons (hiNs) were generated with *BIN1* knock-out (KO).

Lewy body-like inclusions, one of the key features of PD, were observed in the human midbrain-like organoids (hMLOs) derived from GBA1^−/−^ and SNCA overexpressing isogenic ESCs, suggesting that the hMLOs could recapitulate the underlying mechanisms for progressive Lewy body formation [67]. Most regular brain organoids bear the limitations of heterogeneity and long-term differentiation. To overcome these shortcomings, the simplified brain organoids (simBOs) composed of mature neurons and astroglial cells were generated from the hPSC-derived primitive neural stem cells (pNSCs). The midbrain-like simBOs bear several advantages over the traditional brain organoids such as rapid generation, high homogeneity, and easy specification into midbrain-like organoids via treatment with Shh and FGF8. The simBOs generated from a PD patient with a mutation of LRRK2 demonstrated typical symptoms like upregulated LRRK2 activity, down-regulated dopaminergic neurons, and enhanced autophagy. Moreover, treatment of simBOs with PFE-360, an LRRK2 inhibitor, could relieve the abnormalities, suggesting the potentiality of simBOs serving as PD models and alternative platforms for drug-testing and screening [68].

#### 2.2.3. The PARK7-linked PD Organoids

The midbrain organoids derived from the Ibrahim Boussaad1 PD (PARK7-linked PD) patient, a highly heterogeneous neurodegenerative disorder, have been applied for the characterization of aberrant RNA splicing. It turned out that U1 splicing site mutations were enriched in sporadic PD patients, leading to a significant reduction of DJ-1 proteins and causing consequential mitochondrial dysfunction. The organoid-based drug testing has enabled the identification of certain compounds such as phenylbutyric acid as well as the genetically engineered U1-snRNA. These compound hits have been further tested and validated to be effective in reversing mis-splicing, mitochondrial dysfunction, and dopaminergic neuron loss in the mutant midbrain organoids. This could be an efficient alternative strategy for precision medicine to treat sporadic PD by molecularly targeting the splicing abnormality to rectify cellular mitochondrial dysfunction [69].

#### 2.2.4. PD Organoids for Toxicology Study

A robust method has been developed to generate human organoids and incorporate microglia together with astrocytes into the organoids for studying toxicology and pathophysiology of the CNS. This type of organoid has been employed to test the PD model toxicants and will be promising for drug screening in the future [60]. More PD organoid models were established to test and characterize the neurotoxic effects on dopaminergic neurons via a machine learning-based analytical method. This approach has been used for the high content calcium image-based (HCI) cell profiling and toxicity evaluation in midbrain organoids treated with/without 6-OHDA, a neurotoxic compound. This platform could be employed for modeling PD and drug screening to identify the neurotoxic compounds ([70]. Further improvement was made for the generation of midbrain organoids to avoid the inherent shortcomings including batch-to-batch variability and the presence of a necrotic core. The midbrain organoids simulate some key features of midbrain development like dopaminergic neuron and astrocyte differentiation. This strategy is efficiently suitable for pathological studies on toxin-induced PD [71].

The MOSs generated with the improved protocol by Kwak et al., are homogeneous with mature architecture of midbrain dopaminergic (mDA) neurons, other neuronal subtypes, and functional glial cells such as astrocytes and oligodendrocytes. More importantly, these MLOs are extremely sensitive to 1-methyl-4-phenyl-1, -2,3,6-tetrahydropyridine demonstrating mDA neuron-specific cell death. Thus, the MOs could serve as a platform for the in vitro study of PD pathology as well as drug screening for PD therapy [61]. Renner et al. developed midbrain organoids that claimed to be able to faithfully recapitulate the main characters such as architecture, size, cellular composition, homogeneous morphology, aggregate-wide synchronized neural activity, and global gene expression. They then employed the midbrain organoids to create a scalable and HTS-compatible platform in standard 96-well-plates for drug screening and evaluation with the criteria of HCI and RNA-seq at the single-cell level. By automating the entire workflow from generation to analysis, the intra- and inter-batch reproducibility was enhanced as demonstrated by RNA-seq and HCI. It turned out that this platform could be automated to generate the reproducible prediction of the drug effects on neurological disorders such as PD at the single-cell level albeit within a complex organoid environment [72].

#### 2.2.5. Organoids Generated through a Carbon-Based Scaffold for Modeling PD

To overcome necrosis within the organoids during the long-lasting cultures, carbon fibers (CFs) have been employed as a new type of scaffold to generate midbrain organoids in replacement of the conventional polylactide-co-glycolide copolymer (PLGA) scaffold. Physiochemically, the porosity, microstructure, or stability of CF scaffolds could improve efficiency in iPSC differentiation within organoids relative to the PLGA scaffolds. The midbrain organoids generated in the CF scaffolds could more efficiently recapitulate the midbrain development evidenced by the expression of key regulator genes such as *PITX3* for terminal differentiation and the survival of midbrain dopaminergic (mDA) neurons. This strategy is promising for the establishment of the organoids in modeling neurodegenerative diseases associated with the midbrain such as PD and drug screening platforms [59].

### 2.3. Modeling of Fragile X Syndrome (FXS)

Fragile X syndrome (FXS) is one of the NDDs with key features of intellectual disability and sensory deficits caused by a loss of FMRP, a multi-functional RNA binding protein. Compared to the in vitro brain organoid models for other NDDs, so far only three independent research laboratories reported brain organoid models for FXS [6, 73, 74]). Human forebrain organoids were generated from the iPSCs derived from healthy control and FXS patients, respectively, to model FXS in vitro [6]. It turns out that dysregulation was observed in neurogenesis, neuronal maturation, and excitability of the FXS organoids as compared to healthy organoids. A different group has generated cortical organoid models for FXS by knocking out the FMR1 gene [73]. Cellular and molecular tests confirmed the alteration of gene expression, aberrant differentiation, increased number of glial cells, enhanced spontaneous network activity, and depolarizing GABAergic transmission compared to the healthy counterpart. However, so far, the FXS-derived organoids have been not tested for drug screening.

### 2.4. Modeling of ASD Using Brain Organoids Derived from ASD Patients

Autism Spectrum Disorder (ASD) is caused by early neuron developmental dysfunction and lasts for the entirety of life, lacking clear etiology and genetic basis, but is linked to abnormal social communication and behaviors [75, 76] [11, 12, 75, 77, 78]. The incidence of ASD is approximately 1 in 59 children and 1% of the global population according to the CDC statement (https://www.afhu.org/2017/10/23/what-to-know-about-asd/?gclid=EAIaIQobChMI-oD087qQ7gIVh56zCh11og-UEAAYBCAAEgKxmPD_BwE). The organoids derived from ASD patients have been employed for modeling, pathological studies, and drug screening [34, 76].

The telencephalic organoids from the affected families were generated for modeling the idiopathic ASD for the first time using these cerebral organoids. Relative to organoids from the unaffected family members, significant cellular alterations were detected in the ASD organoids including synaptic growth, cell cycle function, and imbalance in GABAergic/glutamatergic neuron differentiation. Molecularly, the altered gene expression network could contribute to the pathogenesis of ASD. For instance, the enhanced expression of FOXG1 leads to the overproduction of GABAergic inhibitory neurons [12]. To investigate the metabolic pathway networks that contribute to ASD pathogenesis, human cerebral organoids were produced to harbor mutations of Rab39b, a small GTPase associated with X-linked macrocephaly, ASD, and intellectual disability. The enhanced proliferation and impaired differentiation of neural progenitor cells (NPCs) were observed in the *RAB39b* mutant cerebral organoids, leading to an enlarged size of the organoids that resemble the trait of ASD. At the molecular level, the interaction between *RAB39b* and PI3K components was confirmed by the promotion of the PI3K–AKT–mTOR signaling in NPCs of the Rab39b mutant cerebral organoids (Figure 1(f)). Furthermore, the enlarged organoid sizes and NPC over-proliferation caused by Rab39b mutation were rescued by the inhibition of AKT signaling, providing a platform to study the pathology of ASD and drug screening [79]. To further investigate the mechanism of ASD at a cellular and molecular level, mouse cortical organoids (mCOs) were generated from the KO of contactin-associated protein-like 2 (CNTNAP2), a member of the neurexin protein family. At the cellular level, defective generation of the GABAergic inhibitory neurons was observed in the KO mCOs. Consistently, at the molecular level, the dysregulated transcriptional network involved in GABAergic neurogenesis was demonstrated at the neural progenitor stage without Cntnap2. Furthermore, the dysregulations in the KO mCOs at the cellular and molecular levels could be rescued by treatment with retigabine, an antiepileptic drug, suggesting that Cntnap2 could serve as a therapeutic target for clinical therapy of ASD [68].

Microduplication at 7q11.23 (7Dup), harboring 26–28 genes, is marked to be a highly associated genetic mutation relevant to ASD. The cortical neurons derived from this microdeletion offer unique opportunities for translational studies at the genetic and pathological levels as well as for drug screening to identify drug efficacy in therapy. Williams–Beuren syndrome (WBS), characterized by hyper sociability and language strengths, is caused by microdeletion with several genes located within the deleted region such as *GTF2I*, *BAZ1B*, *CLIP2* and *EIF4H*. These have been acknowledged as potentially crucial contributors to the pathogenesis of WBS.

The cortical glutamatergic neurons derived from the WBS patients were employed for a large-scale drug screening to identify the hits from a small molecule compound library consisting of potential reagents for CNS, epigenetic modulators, and function-unknown compounds. By comparing the transcriptional alteration of the WBS interval genes, three histone deacetylase inhibitors (HDACi) were identified and further validated at the levels of both mRNA and protein to downregulate the expression of *GTF2I* with a prevalent pathogenic role [80].

More recently, cerebral cortical organoids were generated from iPSCs carrying the mutations in *KMT5B*, *ARID1B,* and *CHD8*, three ASD risk genes or the wild-type genes for modeling of ASD. These organoids were used to identify aberrant cell-type-specific neurodevelopment shared across ASD risk genes and investigate the underlying mechanisms of these genes in their contribution to ASD pathology [81]. Given the complexity of neurological disorders and the limitations of the animal models in the pathological study, human brain organoids will play vital roles in modeling disorders and drug screening procedures [82].

## 3. Advances in Organoid-Based Drug Screening

A workflow for drug screening using serum-free embryoid bodies (SFEBs) derived from hiPSCs for scalable high-throughput screening (HTS) has been developed. The screening was conducted with criteria of multi-electrode arrays (MEAs) to show the firing and burst rates determined by single-cell HCI to assess the number of excitatory neurons, demonstrating a high degree of consistency and reliability. Thus, the SFEBs could serve as a platform for HTS to enumerate the high variation in cortical organoids. Although this strategy is time-consuming, it could serve as an efficient starting point for phenotypic drug screening [83]. The current brain organoids resemble the early stage developing brain; developing brains are more sensitive to toxic exposure relative to fully developed brains [83]. Therefore, the brain organoids could serve as an ideal platform for screening developmental neurotoxicity. Brain organoids have been applied for modeling early-stage neurotoxicity screening. With this platform irreplaceable by in vivo animal models or cell-based screening [84], large-scale chemicals in use and potential drugs in the future could be determined. Thus, this strategy opens a new avenue for evaluating toxicants by determining if members of the compounds library potentially belong to developmental neurotoxicants. Successful studies have been conducted to identify drugs and heavy metal chemicals as developmental neurotoxicants [84–86].

**Figure 2:**
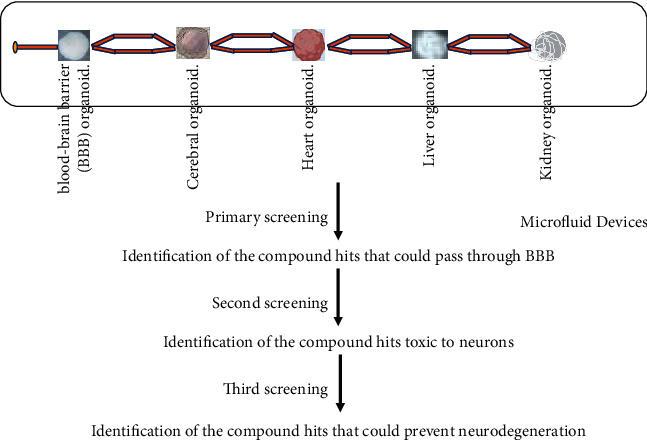
The body-on-a-chip-based drug screening flowchart. The different tissues/organs-specific organoids were arranged in order with BBB organoids in the first place followed by cerebral organoids and other organoids that recapitulate their corresponding tissues/organs in a body-on-a-chip device. Several successive screening processes could be conducted starting from the primary screening to identify the compounds that could pass the blood-brain-barriers (BBB) followed by the second and third rounds of screenings to identify the compound hits that are toxic to neurons and that could rescue neurodegenerations, respectively.

Due to the selective permeability of drugs to the brain, BBB impairment or dysfunction in many types of NDDs contribute to pathogenesis [87–89]. Therefore, the BBB serves as one of the key structures for drug discovery for the therapy of human NDDs [87], indicating a potential first target for new drugs to enter the brain. Most of the current organoid models are single tissue or organ-based, failing to orchestrate multiple different relevant tissues or organs let alone the system levels. Given the evolutionary distance between humans and mice, the discrepancy between BBB function and brain microvascular endothelial cells (BMECs) dampens the simulation of animal models to humans [90]. To mimic the human CNS and circulation system-level interactions, several physiologically relevant BBB-on-a-chip models have been established, composed of brain neural/organoids, the BBB, and a vascular side separated by a porous membrane [146–150], several of which are reported to model drug penetrability accurately [91–93] (Figure 2).

Recently, human CNS barrier-forming organoids (CBFOs) were established from the choroid plexus (ChP), a protective epithelial BBB by which the cerebrospinal fluid (CSF) is produced. The CSF is a vital liquid that provides nutrients and signaling molecules to help remove toxic waste products to aid in the survival and maturation of the brain. ChP selectively permeabilizes entry of the molecules to avoid free entry of toxic molecules or drugs from the blood. The human ChP-CSF organoids recapitulate the main traits of the ChP. On one hand, the ChP-CSF organoids could secrete the CSF-like fluid to mimic in vivo CSF. On the other hand, the ChP-CSF organoids have a restrictive barrier that exhibits the same selective permeability to small molecules in vitro as the ChP in vivo. The ChP-CSF organoids could progressively mature over time under in vitro conditions. Molecularly, ChP-CSF organoids bear a high degree of similarity to the ChP in vivo at the transcriptomic and proteomic levels. Combined transcriptomic and proteomic analysis at the single-cell level leads to the identification of key human CSF components undetected but produced by epithelial subtypes. More importantly, the ChP-CSF organoids can be employed to predict the permeability of new compounds into the CNS [94]. Thus, the new CBFOs-on-a-chip model may successfully simulate the selective permeability of drugs into the brain, thereby functioning as a platform to carry out drug screening for easy translation into clinical therapy of NDDs (Figure 2).

Shortcomings of the current approaches for organoid-based drug screening.

Human organoids have been acknowledged as a relatively ideal versatile tool for modeling human diseases, in vitro pathological studies, and drug screening. However, the current drawbacks at the level of organoids hampered the reliability and the efficiency of drug screening. At the organoid level, the quantity and quality of organoids significantly impact the drug screening efficiency and reproducibility. Due to the low generation efficiency of organoids, the scale of the organoids has become one of the bottleneck limiting factors for the efficiency and reproducibility of the drug screening. In addition to the scale of the organoids, the quality of the organoids including the capacity of simulating their parental organs or disease phenotypes dramatically affect the reliability of the drug screening. At the systemic level, one of the main fatal shortcomings in most organoid models consists of the ignorance of the drug's interaction with multiple tissues/organs/systems in vivo. The issue is particularly important for NDDs due to the existence of the BBB.

## 4. Future Perspectives

Significant achievements have been made in the generation of human organoids, particularly in the brain and cancer-specific organoids. These organoids generated by utilizing current strategies could recapitulate key features of the human brain, making it possible for in vitro studies on neurodevelopment and modeling of NDDs. The organoid-based small- or large-scale drug screening processes proved to be promising with some compound hits being identified and validated for therapy. However, to a larger extent, these brain organoids are incomparable to human brains both architecturally and functionally. Therefore, the generation of organoids and the organoid-based study remain in the infancy stage. Among other issues, overcoming the limitations to generating high-quality organoids has been the top priority. The basic requirement is to enable the organoids to faithfully recapitulate key features of the brain region(s). To effectively mimic the human brain, further characterization and comparison of the human fetal, postnatal, adult, and aging brains at structural, cellular, and molecular levels is indispensable [95–98]. Although the current brain organoids at different ages could partially recapitulate the developing stage of their in vivo brain counterparts, they bear some shortcomings for modeling neurological disorders such as NDDs:

1. Vascularization. Currently, most organoids lack 3D vascular networks limiting neurogenesis, proliferation, differentiation, apoptosis of organoids, and long-term culture, leading to a low efficiency in recapitulating the late stages of human brain development. This issue could be partially ameliorated by vascularization via genetically engineered induction of ETV2, co-culture with epithelial cells, or by graft of human brain organoids into mouse brains. However, the capacity of oxygen, nutrient supply, and metabolic clean-up provided by this alone remains to be insufficient. More recently, several strategies for improving brain organoid vascularization were invented. The first method consists of co-culturing neuronal spheroids with perfusable blood vessels. The vascularized neuronal spheres could efficiently enhance proliferation, differentiation, and reduce apoptosis [99]. The second method is comprised of the separate generation of vessel organoids and brain organoids followed by a co-culture of two types of organoids. Increased number of neural progenitors, functional BBB-like structures, and active microglial cells were observed in the fused/vascularized brain organoids. Therefore, the fused organoids enable us to investigate interactions between immune and non-immune cells as well as neuronal and non-neuronal cells in vitro [100]. Thus, these two strategies could serve as a better tool to simulate brain development and model neurological disorders. However, some concerns remain to be against this strategy. Effectively improving the quality and distribution of vessels in organoids in concert with biomaterials and microfluidic system-based technology could be promising in this regard
2. Most brain organoids usually represent early fetal brain development, whereas some NDDs such as AD and PD are usually late-onset. Thus, the application of organoids for modeling late-onset NDD-associated aging progression such as PD is limited. Fortunately, human cortical organoids could mature to 250~300 days postnatal, parallel to in vivo development and maintenance of in vivo developmental milestones. Furthermore, the genes critically involved in neurodevelopment and NDD risks were mapped to in vitro gene expression trajectories. This suggests that human cortical organoids hold the potential for long-term cultures, which parallels in vivo developmental progression and maturation [101]. Therefore, appropriately maintaining the long-term maturation of human cortical organoids and avoiding necrosis and abnormalities during the culture are essential to generating brain organoids that match key postnatal transitions for modeling NDDs
3. Lacking microglia, the key player in the developing brain, in the current brain organoids has been an essential drawback for modeling NDDs, limiting the application of brain organoids. Co-cultures are the conventional strategy for integrating microglia into brain organoids. Indeed, the human microglia could be integrated into human midbrain organoids [71, 102–104]. Xu et al. developed a new protocol for generating brain region-specific microglia-containing organoids by co-culturing at a proper time point [105]. Bodnar et al. developed a protocol to generate microglia-containing CO (MCO) by a novel technique for embryoid body (EB) production directly from iPSCs combined with orbital shaking cultures. However, the microglia ratio remains low (~7%) [106]. Interestingly, it was observed that during cultures, erythromyeloid progenitors migrating to brain organoids could gradually develop into microglia-like cells [107]. Recently, a protocol was developed for the generation of microglia-containing hCOs (mhCOs) via the overexpression of myeloid-specific transcription factor PU.1 in cortical organoids without co-culture. The mhCOs have become an efficient tool for functional investigation of microglia in neurodevelopmental and neurodegenerative disorders such as AD [108]. Given that microglia could not emerge natively inside organoids using the previous methods, this novel strategy has been a breakthrough for microglia generation in brain organoids. However, further improvement in the protocol is required to maintain native microglia emergence with a controllable microglia ratio. These brain organoids will be essential for modeling NDDs, in vitro pathological studies, and drug screening
4. The limited size and heterogeneity of the current brain organoids offer inefficient representative capacity to their in vivo counterparts. On the other hand, separately generated organoids that represent different brain regions could be assembled to generate whole-brain organoids to recapitulate the entire brain more faithfully as compared to their separate counterparts [109, 110]. Thus, breakthroughs in the generation of the fused organoids open a new window to investigate the crosstalk at the inter- brain-region and the inter-organ levels. However, the assembly of the whole brain organoids is still at the infancy stage; many technical issues need to be resolved such as guiding border formation and interconnection of the separate tissues

Assembloids generated from the co-culture of different brain regions of organoids have been employed to investigate the internal interactions between the brain regions but fails to offer the tool for understanding signal transduction from the brain to the whole body. In addition, assembloids stem from the fusion of human organoids bearing the shortcomings of high heterogeneity and variable reproducibility. Ao et al. developed a simple and versatile acoustofluidic method to partially overcome these disadvantages by a controllable spatial arrangement of organoids [111]. Recently, a breakthrough was made in the generation of engineered brain-spinal cord assembloids (eBSA) by co-culturing cerebral organoids (COs) and motor neuron spheroids (MNSs) [100]. The eBSA connects COs and MNSs to recapitulate the brain-spinal cord connection. Potentially, the eBSA could serve as a platform to screen and validate neurochemical stimulus signal transduction. In addition, the accumulation of knowledge regarding the neural signal transfer from the CNS to the peripheral nerve system (PNS) will provide a better understanding of controlling muscle actuators within the nervous system.

(5) Under in vitro culture conditions, the growth and maturation of the brain organoids are time-consuming. To overcome this issue, pharmacological strategies have been proposed for accelerating growth and maturation. However, these pharmacological strategies may potentially result in the alteration of intrinsic differentiation processes programmed naturally, interfering with the recapitulation of the resulting brain organoids to their in vivo counterparts. Efforts have been made to genetically induce aging [112–114]. However, these genetic operations should be further improved and validated to determine if the resulting brain organoids are reliable in terms of faithfully modeling pathological features, particularly disease-associated aging
(6) Large scale drug screening has been carried out in cancer organoids, and some compounds have been successfully identified for further assessment and validation. However, limited information is available for the brain organoid-based high throughput drug screening, but some previously tested compounds and current clinical drugs were tested in brain organoids that could model AD, PD, and ASD, respectively. Key issues for brain organoid-based drug screening is reliability and efficiency, particularly for the organoids for NDD modeling. Many factors affect the efficiency and reliability of brain organoid-based drug screening. Currently, most drug tests were conducted on the region-specific organoids. The variability of the organoids derived by the self-organization of neuronal cells hinders the efficiency, reliability, and availability of personal medicine. The whole-brain organoids assembled from separately generated organoids representing different brain regions could be more reliable for drug screening. The recent development of the BBB organoids could efficiently prescreen the permeability of new compounds passing through the BBB first before functional screening for a potential therapy for neurological disorders

**Figure 3:**
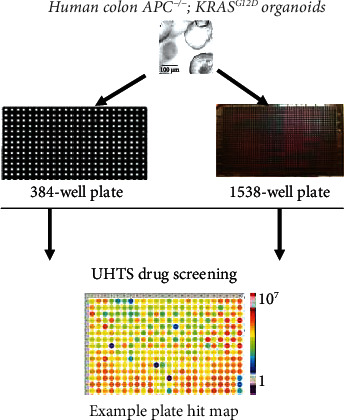
The cryo-preserved human colon organoids with APC^−/−^; KRAS^G12D^ mutation could confer the long storage and re-grown upon cryo-recovery for expansion to make the culture operation alike for the cell lines. This strategy could significantly overcome the bottleneck limitation of the organoid supply for the ultra-high-throughput screening (UHTS) in 384-well and 1538-well plates.

Another issue for large-scale drug screening using cerebral organoids is developing long-term storage and culture operations alike for the cell lines. Although no cerebral organoids were reported to have this property, success of the colon *APC^−/−;^ KRAS^G12D^* organoids [115] shed light on the development of cerebral organoids with this property (Figure 3). It is highly expected that the brain organoids with long-term storage and culture operations alike for the cell lines will be developed in the future.

As a summary, the establishment of organoids has been a milestone for the in vitro modeling of in vivo organ development, pathological studies, and drug screening albeit numerous difficulties remain to be resolved. The rapid and comprehensive progress in the organoid technologies shed light on the future breakthrough in overcoming the inaccessibility of human organs/systems via in vitro organoid-based platforms. However, being mindful that in vitro models cannot perfectly mimic in vivo counterparts will inspire investigators to make efforts to improve the technology and research strategies.

## Figures and Tables

**(a):** 

Culture medium/supplement/culture strategy	Regions/organoids type	Key features	References
SHH, FGF8	Midbrain simBOs	High efficiency, high homogeneity, easy to specify	[58]
CHIR99021	Midbrain-like MLOs	Robust generation Homogenous distribution of mDAs, other neuronal subtypes, and functional glial cells, such as astrocytes and oligodendryocytes	[61]
WNT3A and mixed medium with 1 : 1 of fresh and supernatant derived from interfollicular epidermal SCs	Epidermal organoids	Functional with polarity	[120]
RSPO1, WNT3A, WNT7A	Endometrial organoids	Endometrial disease facilitate growth of endometrial disease organoids and the long-term expansion	[121]
WNT and nodal antagonists	Hippal/CB organoids	Original differentiation method Low efficiency of O2 and nutrient diffusion	[122]
Dkk1 and LeftyA			
Floating culture with 40% O2 and 5% CO2 CHIR 99021, BMP4	Hipp/cortex organoids		[10, 13, 24,119–121]

**(b):** 

Culture device	Key features	References
1. Spinning bioreactor	High cost and require a high volume of culture medium	[122]
2. Multiple-well culture plates with orbital shakers	Reducing the cost and consumption of the culture medium successful generation of cerebral organoids	[123, 124]
3. Miniaturized multiwell spinning bioreactor	Facilitate the establishment of brain region-specific organoids that mimic the dorsal forebrain, midbrain, and hypothalamus	[14, 95]
4. Collagen hydrogel systems	Consisting of interconnected excitatory and inhibitory neurons with supportive astrocytes and oligodendrocytes fiber for bioengineered organoids A highly interconnected neuronal network established in organoids at a macroscale tissue format.	[8]
	More importantly, the engineered organoids share structural and functional similarities with the fetal brain, potentially allowing for the study of neuronal plasticity and modeling of disease	
5. Carbon fibers (CFs) for midbrain organoids	The porosity, microstructure, or stability CF scaffolds could improve efficiency in iPSC differentiation within organoids relative to the PLGA scaffolds. The midbrain organoids generated in the CF scaffolds could more efficiently enhance terminal differentiation and the survival of midbrain dopaminergic (mDA) neurons.	[59]
6. Brain organoids	The modified hydro-Matrigel with an interpenetrating network (IPN) of alginate has been employed to maintain the mechanical microenvironment for brain organoids, conferring the viable growth environment with the characteristic formation of neuroepithelial buds.	[125, 126]
7. Brain organoids	The platform of “tissue-like” cyborg stretchable mesh nanoelectronics were invented to provide seamless and noninvasive coupling of electrodes to neurons within developing brain organoids, enabling continuous recording of single-cell action without interruption to brain organoid development	[127]

**(c):** 

Coculture of organoids	Key features	References
Co-culture of cancer organoids with other non-tumor cells	Tumor organoids could get other cell types of cells and tissues	[128, 129]
Vascularization of organoids		
1. Direct transplantation of the brain organoids into mouse brains		[32, 34, 130]
2. Coculture of brain organoids with epithelial cells followed by transplantation into mouse brains		[131]
3. Genetic operation-based vascularization	Expression of human ETS variant 2 (ETV2) in human cortical organoids (hCOs), led to generation of the functional vascular-like vessels in the vascularized hCOs (vhCOs), improving organization, alleviating hypoxia, and reducing apoptosis	[132]
4. BVO cells infiltrate into brain organoids	High efficiency to generate vascularized human brain organoids	[133]
5. The microfluidic chips-based coculture with epithelial cells		[134]
6. Vascularized spheroid using an injection-molded microfluidic chip	By coculturing the spheroids derived from induced neural stem cells (iNSCs) with perfusable blood vessels, the vascularized spheroid was generated. The vascularized spheroid network significantly improved spheroid differentiation and reduced apoptosis.	[99]

**(d):** 

Differentiation methods		
Unguided strategy	Generation of brain organoids with mixed cell lineages of forebrain, midbrain, hindbrain, and retina, enabling the organoids to grow with minimum external interference High variability and heterogeneity	[11, 31, 95]
Guided strategy	Directed differentiation to generate brain region-specific organoids, such as cerebral cortex, hippocampus, midbrain, and cerebellum	[10, 13, 14, 119, 135, 136]
Fused culture technologies for integration of different regions of the organoids	More closely resembling the complexity of the brain in identity, architecture, and interaction manners enhanced the formation of microcircuits with the local excitatory neurons	[123, 124, 135]
Long-term propagation, storage, and regrowth following the frozen and thaw cycles	CRISPR-Cas9-based knock-in of the mutant *KRAS^G12D^* allele into human colon *APC^−/−^* organoids	[115, 137]
Application of 3D printing technology in	Enabled an engineered organ to maintain the spatial arrangement	[39, 134, 138, 139]
Organoids-on-a-chip based approach to	Could remove the dead cells via connecting with an external pumping	[140]
Generate the tube-shaped epithelial organoids	System, extending tissue lifespan and enabling the colonization of organoid tubes with microorganisms to model the host–microorganism interactions	
Generation of microglia cell-containing microglia cerebral organoids	Microglia were naturally developed in cerebral organoids and displayed similar characteristic ramified morphology as in normal fetal brains.	[106, 141]
Generation of microglia-containing hCOs (mhCOs)	Microglia-containing hCOs (mhCOs) were generated via overexpression of the myeloid-specific transcription factor PU.1 in cortical organoids. The mhCOs have become an efficient tool for functional investigation of microglia in neurodevelopmental and neurodegenerative disorders, such as AD	[108]

**Table 2:** Current brain organoids for modeling of neurological disorders.

Organoid type/brain regions	Disease	References	Main discovery
Human forebrain organoid	FXS organoids with	[6]	Dysregulated neurogenesis, neuronal maturation, and neuronal excitability in the forebrain loss of FMRP. Inhibition of the PI3K signaling could rescue developmental deficit of the FXS forebrain organoids
Cortical brain organoid cortical organoids	FXS	[73]	Increased number of glial cells, and bigger organoid size compared to normal person
Cortical brain organoid	FXS	[74]	FXS organoids bear higher percentage of Ki67^+^SOX2^+^ proliferative cells PI3K functions as a key driver of downstream dysregulation of both translations and cell proliferation in early NPCs.
Cerebral organoid	ALS/FTD	[142]	Recapitulates mature cortical architecture and early molecular pathology of C9ORF72 ALS/FTD. Increased levels of the autophagy signaling protein P62 in astroglia. Accumulation of DNA damage, poly(GA), and nuclear pyknosis in deep layer neurons
Sensorimotor organoid	ALS	[143]	Diversity of neuronal derivatives, such as motor, sensory neurons, astrocytes, and mesodermal derivatives, including vasculature, microglia, and skeletal muscle. The NMJs connect the motor neurons and skeletal muscle, but the NMJs were defected in ALS organoids. Altered ability for deriving the NMJ synapse and cell diversity that exert autonomous and noncell autonomous effects on motor neurons
Schizophrenia (Scz) cerebral		[144]	In the Scz organoids, the progenitor survival significantly changed led to disruption of neurogenesis, ultimately generating fewer neurons within developing cortical fields compared to the normal organoids.
Cerebral organoids (iCOs)	AD	[145]	Miniaturized AD pathological models and CRISPR-Cas9-edited isogenic lines established a high-content screening (HCS) system, and the FDA-approved drugs were tested for the blood–brain barrier-permeability
Cerebral organoids whole brain	AD	[38]	The organoids from patients affected by familial AD or DS displayed pathological features of AD, such as accumulation of structures like amyloid plaques and neurofibrillary tangles, but nondetectable in the control organoids.
Cerebral organoids whole brain	AD	[53]	Significant apoptosis, impaired synaptic integrity, enhanced stress granules and disrupted RNA metabolism were detected in cerebral organoids (CO) with *APOE ε*4/*ε*4 genotype from AD patients. Conversion of *APOE4* to *APOE3* ameliorated the *APOE4*-associated phenotypes in Cos from AD patients. *APOE4*-related degenerative pathways were assumed to contribute to AD pathogenesis.
Cerebral organoids whole brain	AD	[56]	CKD-504, a highly BBB-penetrating HDAC6 inhibitor, significantly reduced tau via acetylation in AD patient-iPSC-derived brain organoids, dramatically attenuating pathological tau and ultimately rescuing the synaptic pathologies
Cerebral organoids whole brain	AD	[48]	Cerebral organoids (Cos) generated from PITRM1-KO iPSCs recapitulated AD pathological features such as the accumulation of protein aggregates, tau pathology, and neuronal cell death. ScRNA-seq discovered mitochondrial function defect in all cell types in COs with PITRM1-KO. PITRM1-linked neurodegeneration caused by defects of mitochondrial presequence processing induce an early activation of UPRmt, supporting a mechanistic link between mitochondrial function and common neurodegenerative proteinopathies.
Cerebral organoids whole brain	AD	[46]	Compared with the isogenic control organoids, AD organoids with PSEN2N141I mutation recapitulated an AD-like pathology at the molecular, cellular, and network level, such as a higher A*β*42/A*β*40 ratio and enhanced neuronal hyperactivity. Altogether suggests these isogenic organoids as a promising tool for the pathological study of AD.
Cerebral organoids whole brain	AD	[54]	An episomal plasmid vector derived from EBV based simple and versatile genetic engineering was employed to efficiently generate organoids harboring a normal tau protein with fluorescent tag vs. a mutant genetic form (P301S) of tau that leads to fronto-temporal dementia. The harbored plasmid did not affect differentiation, and the isogenic organoid lines were stable for more than 30 passages expressing either normal or mutant form. The cerebral organoids manifested hyperphosphorylation of the tau protein, a pathologically relevant phenotype, contributing to disease modeling, personalized medicine and potentially translating to clinical therapeutics.
Cerebral organoids whole brain	AD	[55]	The enhanced spontaneous action potentials, slow oscillatory events (~1 Hz), and hypersynchronous network activity were detected in the AD neuronal organoids. The dual-allosteric NMDAR antagonist NitroSynapsin, revoked the hyperactivity, but the FDA-approved drug did not, suggesting the AD organoid models could be efficient tool for drug screening and modeling of the related synaptic damage in AD.
Cortical organoids cortex	AD	[51]	Time and spatial patterns of tau expression at a molecular level was compared during brain development using the iPSC-derived cortical organoids and developing human brains. Neuronal maturation led to the dramatic elevation of tau mRNAs, while low expression levels were observed in SVZ radial glia and deep white matter intermediate progenitors. This system could help further study on the pathophysiological mechanism of triggering and enhancing tau expression, simplifying the identification of therapeutic targets for tauopathy-based neurodevelopmental disorders.
Human midbrain-like organoids (hMLOs)	Early-onset PD	[66]	DNAJC6 mutation vs. CRISPR-Cas9 manifestation of key PD features, pathologic neurodevelopment defects, DNAJC6- mediated endocytosis defect, impairment of the WNT-LMX1A signal during the mDA neuron development reduced *LMX1A* expression during development, generation of vulnerable mDA neurons
Midbrain organoids	PD	[57]	The first organoid model for an idiopathic form of PD and healthy volunteers were generated by the Sendai viral vector mediated transduction. The mature organoids manifested statistical differences in the expression levels of neuronal early and late markers between organoids from PD patient and healthy volunteer. Altogether suggests that PD human organoids could be potentially suitable for modeling PD and cellular interactions within the human brain.
Midbrain organoids	PD	[63]	Isogenic 3D midbrain organoids with or without a PD-associated LRRK2 G2019S mutation recapitulate the pathological hallmarks observed in patients with LRRK2-associated PD. The protein-protein interaction network in mutant organoids revealed that TXNIP, a thiol-oxidoreductase, is essential for development of LRRK2-associated Parkinson's disease in a 3D environment. Altogether suggests the potential of 3D organoid for modeling sporadic PD in advancing therapeutic discovery.
simBOs	PD	[58]	Simplified brain organoids (simBOs), composed of mature neurons and astroglial cells were rapidly generated in 2 weeks and have more homogeneous properties. The SimBOs facilitates the conversion of pNSCs to mature neuronal systems in the context of neurotransmitter release, synaptic vesicle formation, ion channels, calcium signaling, axonal guidance, extracellular matrix organization, and cell cycle. The simBOs could easily be specified into midbrain-like simBOs by treatment with Shh and FGF8. Midbrain-like simBOs from a PD patient (LRRK2G2019S)-derived pNSCs manifested disease-associated phenotypes in terms of increased LRRK2 activity, decreased dopaminergic neurons, and increased autophagy. Treatment with the LRRK2 inhibitor, PFE-360, relieved the phenotype of Parkinson's disease in midbrain-like simBOs. Taken together, these approaches could be applied to large-scale disease models and alternative drug-testing platforms.
Midbrain organoids	PD	[69]	The patient-based midbrain organoid model of PARK7-linked PD was created, and aberrant U1-dependent splicing was detected, causing a drastic reduction in DJ-1 protein and, consequently, mitochondrial dysfunction. Targeting defective exon skipping with genetically engineered U1-snRNA recovered DJ-1 protein expression in neuronal precursor cells and differentiated neurons. Combinatorial treatment with the small-molecule compounds rectifier of aberrant splicing (RECTAS) and phenylbutyric acid, could restore DJ-1 protein and mitochondrial dysfunction in patient-derived fibroblasts as well as dopaminergic neuronal cell loss in mutant midbrain organoids. Therefore, this system could become an alternative strategy to restore cellular abnormalities in in vitro models of PD and provides a proof of concept for neuroprotection based on precision medicine strategies in PD.
Midbrain organoids	PD	[59]	The physicochemical properties of carbon fibers (CFs) scaffolds make CFs more advantageous over the conventionally applied PLGA scaffold in improving the efficiency of iPSC differentiation within organoids. The organoid generated using CFs scaffolds were used for screening genes that expressed during the organoids differentiation at crucial stage of brain development. Correlation between PITX3, one of the essential factors for terminal differentiation and the survival of mDA neurons, and TH gene expression was detected. Thus, it is plausible to suggest that organoids containing mDA neurons formed on CFs could be suitable for investigation of the midbrain-associated NDD such as PD.
Midbrain organoids	PD	[60]	A fast and robust method to generate human midbrain organoids and incorporate microglia together with astrocytes into the organoids. These ratio-defined and three cell type-based organoids are suitable for the study on toxicology and pathophysiology of the CNS.
Midbrain organoids	PD	[70]	A midbrain PD organoid model was generated and applied to test and characterize the neurotoxic effect on dopaminergic neurons via a machine learning-based analytical method. This approach has been used for HCI cell profiling and toxicity evaluation in midbrain organoids treated with/without 6-OHDA, the neurotoxic compound. This platform could be employed for modeling PD and drug screening to identify the neurotoxic compounds
Midbrain organoids	PD	[61]	The homogeneous midbrain-like organoids (MOs) were generated with mature architecture of midbrain dopaminergic (mDA) neurons, other neuronal subtypes, and functional glial cells such as astrocytes and oligodendrocytes but no microglias. The MLOs are extremely sensitive to 1-methyl-4-phenyl-1,-2,3,6-tetrahydropyridine that conferred the mDA neuron-specific cell death.
Midbrain organoids	PD	[72]	The midbrain organoids generated by Renner et al., could recapitulate architecture, size, cellular composition, homogeneous morphology, aggregate-wide synchronized neural activity, and global gene expression. These midbrain organoids have been employed to create a scalable and HTS-compatible platform for drug screening and evaluation with criteria of HCI and RNA-seq at the single-cell level, generating the reproducible prediction of the drug effects on neurological disorders of PD.
Cerebral organoids	Schizophrenia	[21]	Cerebral organoids of four controls and three schizophrenia patients to model the first trimester of in utero brain development. It was found that progression of the cortical malformation was associated with aberrant FGFR1 signaling
Forebrain organoids	Schizophrenia	[22]	Schizophrenia patient derived forebrain organoids to model human brain development. It was found that disrupting DISC1/Ndel1 complex formation contributes to brain development of schizophrenia patient
Telencephalic organoids	ASD	[12]	The cerebral telencephalic organoids generated from affected families were utilized for modeling the idiopathic ASD for the first time with organoids from the unaffected family members as control. Molecularly, the altered gene expression network could contribute to the pathogenesis of ASD such as the enhanced expression of FOXG1, which leads to overproduction of GABAergic inhibitory neurons. Cellularly, the synaptic growth, cell cycle, and imbalance in GABAergic/glutamatergic neuron differentiation were significantly altered in the ASD organoids.
Cerebral organoids	ASD	[34]	Human cerebral organoids carrying the mutations of Rab39b, a small GTPase associated with X-linked macrocephaly, ASD, and intellectual disability, respectively. Cellularly, the proliferation of NPCs was promoted but the differentiation was impaired in the RAB39b mutant cerebral organoids, and ultimately the size of the organoids, whereby resembling the trait of ASD. These organoids have provided a cellular and molecular platform to study the pathophysiology of ASD and drug screening.
Cerebral organoids	ASD	[68]	Cortical organoids (mCOs) from CNTNAP2 KO mouse dysregulated generation of the GABAergic inhibitory neurons at cellular level and the transcriptional network involved in GABAergic neurogenesis at molecular level. And the dysregulations could be rescued by treatment with retigabine, an antiepileptic drug, indicating the potential Cntnap2 as a therapeutic target for clinical therapy of ASD
